# Efficacy of Functional Remediation on Cognitive and Psychosocial Functioning in Patients with Bipolar Disorder: Study Protocol for a Randomized Controlled Study

**DOI:** 10.3390/brainsci13050708

**Published:** 2023-04-24

**Authors:** Vivian Accardo, Stefano Barlati, Anna Ceraso, Gabriele Nibbio, Eduard Vieta, Antonio Vita

**Affiliations:** 1 Department of Mental Health and Addiction Services, ASST Spedali Civili of Brescia, 25123 Brescia, Italy; 2 Department of Clinical and Experimental Sciences, University of Brescia, 25123 Brescia, Italy; 3 Hospital Clinic, Institute of Neuroscience, University of Barcelona, IDIBAPS, CIBERSAM, 08036 Barcelona, Spain

**Keywords:** bipolar disorder, cognition, cognitive enhancement, cognitive remediation, functional remediation, functioning, psychosocial interventions, psychological therapy

## Abstract

Background: Neurocognitive impairment is a prominent characteristic of bipolar disorder (BD), linked with poor psychosocial functioning. This study’s purpose is to evaluate the effectiveness of functional remediation (FR) in enhancing neurocognitive dysfunctions in a sample of remitted patients with diagnosis of BD in comparison to treatment as usual—TAU. To quantify the neurocognitive damage, the Brief Assessment of Cognition in Affective Disorders (BAC-A) will be used, and the overall psychosocial functioning will be measured with the Functioning Assessment Short Test—FAST. Methods: The randomized, rater-blinded, controlled study will include two arms (1:1) encompassing 54 outpatients with diagnosis of BD-I and BD-II, as defined by the DSM-5 criteria. In the experimental phase, remitted patients aged 18–55 years will be involved. At the baseline, at the end of intervention and at the 6-month follow-up, patients will be evaluated using clinical scales (Young Mania Rating Scale (Y-MRS) and Hamilton Depression Rating Scale (HAM-D)). Neurocognitive measurements and psychosocial functioning will be valued, respectively, with BAC-A and FAST. Discussion: The primary expected outcome is that following FR intervention, patients will exhibit improved cognitive abilities and psychosocial outcomes compared to the participants in the TAU group. It is now recognized that neurocognitive deficits are potential predictors of functional outcome in patients with BD. In recent years, there has been a growing interest in the implementation of interventions that, in addition to symptomatic remission, are also aimed at neurocognitive dysfunctions in order to achieve a recovery of psychosocial functioning.

## 1. Background

Bipolar disorder (BD) is a chronic, recurrent and disabling psychiatric disorder [1,2]. It is characterized by the presence of episodes of mania, hypomania and depression, with euthymic intervals [3], and it is classified as a major medical cause of disability [4], affecting approximately 2.4% of the world population [5].

Cognitive dysfunction is a major feature of BD that is strongly associated with patients’ functional outcome, and several meta-analyses have reported that the most affected cognitive domains are sustained attention, verbal memory and executive functions [6,7,8]. Cognitive deficits in BD are important determinants of overall psychosocial functioning since they are present during the euthymic phases [9,10,11]; in fact, patients frequently experience persistent residual symptoms, cognitive impairment, problems in psychosocial functioning and poor quality of life even outside of mood episodes [12,13]. Given the close link between neurocognition and psychosocial functioning [14], the main target of intervention is complete functional recovery, with a focus on current well-being; it is now established that the quality of life depends not only on clinical remission but also on functional outcomes [15].

As concerns the great clinical variability observed in BD, a growing body of evidence confirms a significant heterogeneity in cognitive performance [16,17,18]. Recent evidence attests the existence of subgroups of patients characterized by different levels of cognitive functioning: some patients show normal cognitive performance, some present a moderate cognitive impairment and others suffer from severe neurocognitive deficits [16,17,19,20,21,22]. In fact, 40% of patients diagnosed with BD show severe deficits in various cognitive domains and 30% show moderate and specific difficulties, while others are cognitively intact [8,16]. As regards psychosocial functioning, some evidence also showed a relationship with social cognitive performance [23].

Considering that cognitive deficits could represent an important target of intervention as they worsen the functional outcome, identifying a correct trajectory of intervention and personalizing programs for the treatment of cognitive difficulties, thus leading to an improvement in functioning, quality of life and well-being, represent central objectives for patients with BD [13]. Although pharmacological treatment is essential in the clinical management of BD and represents the basis for treatment success [24], the need for non-pharmacological interventions is increasingly emerging. Given that, as often happens in BD, the prognosis may worsen due to poor medication adherence [25], an integrative approach could represent a valid solution.

BD involves considerable therapeutic complexity; to implement a successful treatment focusing on clinical remission and on functional recovery, aiming at the patient’s well-being and a better quality of life is advisable [13,15]. A growing body of evidence confirms the importance of integrated treatments that include psychosocial interventions and that facilitate the consolidation of symptomatic improvements induced by drug treatment, thus increasing the quality of life and contributing to a general recovery of psychosocial functioning [26].

Cognitive remediation (CR) is an evidence-based intervention originally designed for patients diagnosed with schizophrenia, with an effectiveness profile that is now well-established and supported by a solid body of evidence [27,28,29]. It is currently the psychosocial intervention with the highest degree of recommendation for treating cognitive impairment in schizophrenia according to recent international guidance [30].

It has also recently received new attention for its potential as an effective treatment for people living with BD, with an adaptable program; comparative studies have reported a similar level of heterogeneity of cognitive impairment in schizophrenia and BD, and considering the significant overlaps between the neurobiological and clinical data available, similar trajectories and common deficits can be observed [31].

However, a current and noteworthy criticism is that CR interventions lack specificity for BD, as the profiles of cognitive deficits in BD and schizophrenia are similar but not identical and are generally less severe in BD [32,33]. Therefore, it appears that programs designed for people living with schizophrenia are perceived as too simple for patients with BD, thereby invalidating their participation and causing scarce adherence [34]. Hence, there is a need to implement more specific interventions for BD that take into account the cognitive heterogeneity of this condition. Particular attention should also be given to the cognitive subgroups with the aim of developing neurocognitive approaches suitable for the different needs expressed.

A stimulating debate has recently been launched with the participation of numerous BD experts, focusing on the real usefulness of a CR intervention that is specifically designed for BD [35,36,37]. Although it is recognized that many patients do not present objectively defined cognitive impairment, it is important to keep in mind that some subjects complain of difficulties that are clinically relevant but are configured as subjective cognitive disorders [36]. According to Burdick and colleagues, this could reflect the presence of unresolved mood symptoms, reversing the idea of interventions aimed solely at cognitive improvement: this issue further highlights the need to implement new interventions for BD [36]. Experts from the International Society for Bipolar Disorders (ISBD) have identified a number of methodological challenges for designing treatments targeting cognitive disorders in BD with the aim to achieve functional recovery [8,38].

Given that, to date, the link between neurocognitive and real-world functioning is not completely clear for people living with BD, it is necessary to further investigate the functional implications deriving from the efficacy of the treatment on cognition. It is worth mentioning that the methodological recommendations for cognition trials provided by the Cognition Task Force from the ISBD encourage the inclusion of a functional measure as a co-primary key measure of functional change [38]. In this regard, in the last decade, there has been a proliferation of psychological interventions specifically aimed at the recovery of psychosocial functioning in BD, with an emphasis on cognitive functioning [37,39,40,41].

In particular, an interesting and innovative program is functional remediation (FR), an intervention specifically developed by the Bipolar and Depressive Disorders Unit of the Hospital Clinic of Barcelona that aims to ameliorate functional outcomes by targeting neurocognitive difficulties in euthymic patients diagnosed with BD [39]. FR is an intervention delivered in a group format and based on the neurocognitive behavioral model that aims to enhance neurocognition and functional impairment associated with BD. It is based on the concept that improving cognitive performance in BD could produce consistent gains in psychosocial functioning and draws upon recent evidence showing that CR and cognitive behavioral interventions are feasible and effective in producing significant cognitive gains in people living with BD [39,40,41,42,43,44,45,46].

FR efficacy was explored in a multicentric, randomized, rater-blind trial, which showed an improvement in general psychosocial functioning, especially in the interpersonal and occupational domains [39]. FR has been shown to be effective in enhancing psychosocial functioning in both BD I and BD II [47,48], and the durability of its efficacy was attested at the one-year follow-up, also showing a persistent improvement in verbal memory [12].

However, more scientific evidence is currently required to replicate the results of FR effectiveness on functional outcomes in clinical settings and to explore its effectiveness on secondary outcomes such as cognitive performance and, in particular, social cognition performance. Moreover, moderators and predictors of response remain to be assessed.

### Trial Aims and Objectives

The present project aims to evaluate the efficacy of FR in improving psychosocial functioning, cognitive deficits and the quality of life in a sample of euthymic patients diagnosed with BD. This intervention will be compared with a control condition: a group of participants will follow a treatment as usual (TAU) program that includes only pharmacological treatment according to good clinical practice.

The primary objective measure is the score on the Functioning Assessment Short Test (FAST) from the baseline to the endpoint. The hypothesis is that an integrated treatment (FR + TAU) may be more effective than the standard treatment (TAU) in improving psychosocial functioning in BD. We hypothesize that patients involved in an FR group will show improvements in overall psychosocial functioning compared to patients included in the control group (TAU).

One secondary objective is to evaluate whether integrated treatment (FR + TAU) can allow a more effective response in improving neurocognitive and socio-cognitive performance and subthreshold affective symptoms compared to standard treatment (TAU). A further secondary objective is the identification of possible sociodemographic, clinical, cognitive and functional predictors of clinical, cognitive and functional responses in the two groups of patients.

## 2. Methods

### 2.1. Trial Governance

This trial is currently registered with ClinicalTrials.gov (NCT04577508) and has been approved by the local Ethics Committee (reference NP 3976, NP 3977 and NP 3978); all the procedures comply with the ethical principles of the Declaration of Helsinki [49] and are of good clinical practice [50]. The Consolidated Standards of Reporting Trials (CONSORT) guidelines [51] will be followed.

### 2.2. Trial Design and Setting

**Figure 1:**
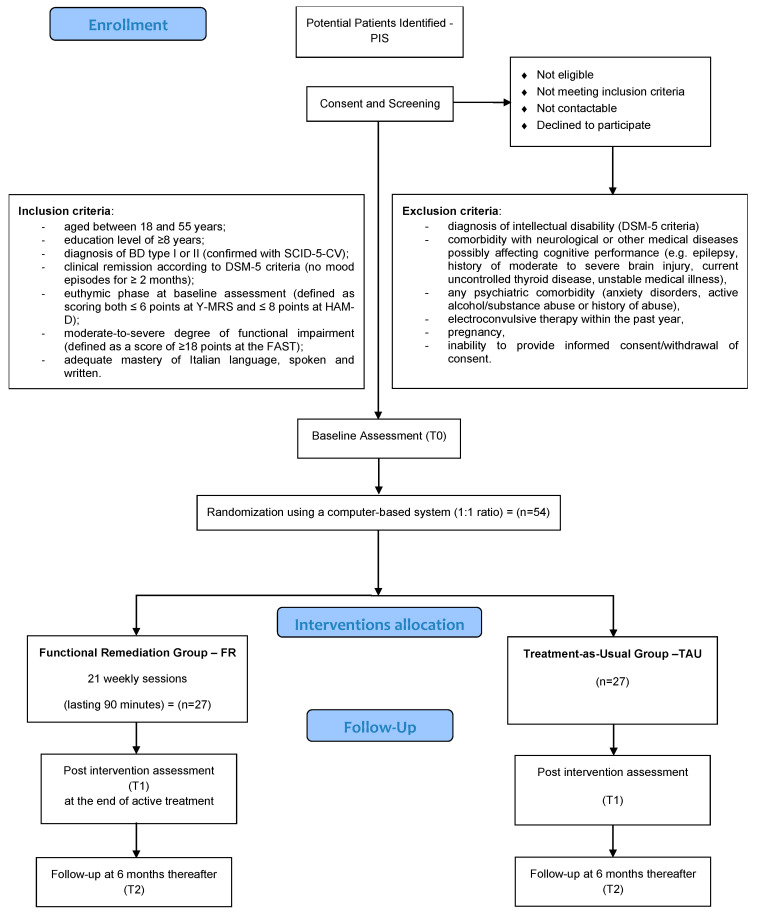
Participant flow chart. Brief Assessment of Cognition in Affective Disorders—BAC-A; bipolar disorder—BD; Diagnostic and Statistical Manual of Mental Disorders—DSM-5; functional remediation—FR; Functioning Assessment Short Test—FAST; Hamilton Depression Rating Scale—HAM-D; Mayer–Salovey–Caruso Emotional Intelligence Test—MSCEIT; Potential Patients Identified—PIS; Structured Clinical Interview for DSM-5 Disorders, Clinical Version—SCID-5-CV; treatment as usual—TAU; Young Mania Rating Scale—Y-MRS.

This is a pilot randomized and rater-blind controlled trial involving 54 clinically stable subjects with a diagnosis of BD type I or II and comprising a 6-month intervention phase and a 6-month follow-up period. Study participants will be assigned to either FR in addition to TAU (n = 27) or TAU alone (n = 27). Clinical assessments will be conducted at baseline, post-treatment and after a follow-up of 6 months (shown in Figure 1).

### 2.3. Participants

Study participants will be recruited from outpatient services or semi-residential or residential care within three different operative units of the Department of Mental Health and Addiction Services of ASST Spedali Civili, Brescia, Italy. In this setting, in line with clinical guidelines [51], standard treatment for subjects with a diagnosis of BD consists of pharmacological treatment and individual case management. The trial will not directly impact concomitant routine treatment. However, patients receiving psychosocial interventions potentially active on cognition will be excluded from the study.

#### 2.3.1. Inclusion Criteria

Eligible participants will be fully informed about the study procedure and included only if providing written informed consent. They will be required to meet the following inclusion criteria: (a) age between 18 and 55 years; (b) education level of ≥8 years; (c) diagnosis of BD type I or II as referred from treating psychiatrist and confirmed at recruitment using the Structured Clinical Interview for Disorder for DSM-5, Clinical Version (SCID-5-CV) [52]; (d) clinical re0mission according to DSM-5 criteria (no mood episodes for ≥2 months) [53]; (e) euthymic phase at baseline assessment (defined as scoring both ≤6 points on the Young Mania Rating Scale (Y-MRS) [54] and ≤8 points on the Hamilton Depression Rating Scale (HAM-D)) [55]; (f) moderate to severe degree of functional impairment (defined as a score of ≥18 points on the Functioning Assessment Short Test (FAST)) [56]; (g) adequate mastery of Italian language, spoken and written.

According to these criteria, participants will not be pre-screened for neurocognitive impairment since the main focus of FR is functioning rather than cognition, and this program is primarily (though not exclusively) aimed at promoting psychosocial functioning among patients with significant impairment in occupational and interpersonal domains [15].

Concomitant medications will be kept stable as much as possible throughout the trial in order to reduce confounding effects. For patients receiving lithium therapy, the serum dose will be monitored over time and maintained within the therapeutic range. No major restrictions will be applied in terms of medication doses or compounds in order to not affect trial feasibility and the generalizability of the results; however, subsequent analyses are going to include the investigation of the potential influence of these parameters on treatment effect.

#### 2.3.2. Exclusion Criteria

Exclusion criteria for the present study are as follows: (a) diagnosis of intellectual disability (DSM-5 criteria); (b) comorbidity with neurological or other medical diseases possibly affecting cognitive performance or the execution of neuropsychological tests (e.g., epilepsy, history of moderate to severe brain injury, current uncontrolled thyroid disease, unstable medical illness); (c) any psychiatric comorbidity (including anxiety disorders, active alcohol/substance abuse or history of abuse in the 3 months prior to screening); (d) electroconvulsive therapy within the past year; (e) pregnancy; (f) inability to provide informed consent/withdrawal of consent.

### 2.4. Procedures

#### 2.4.1. Screening and Randomization Phase

**Figure 2:**
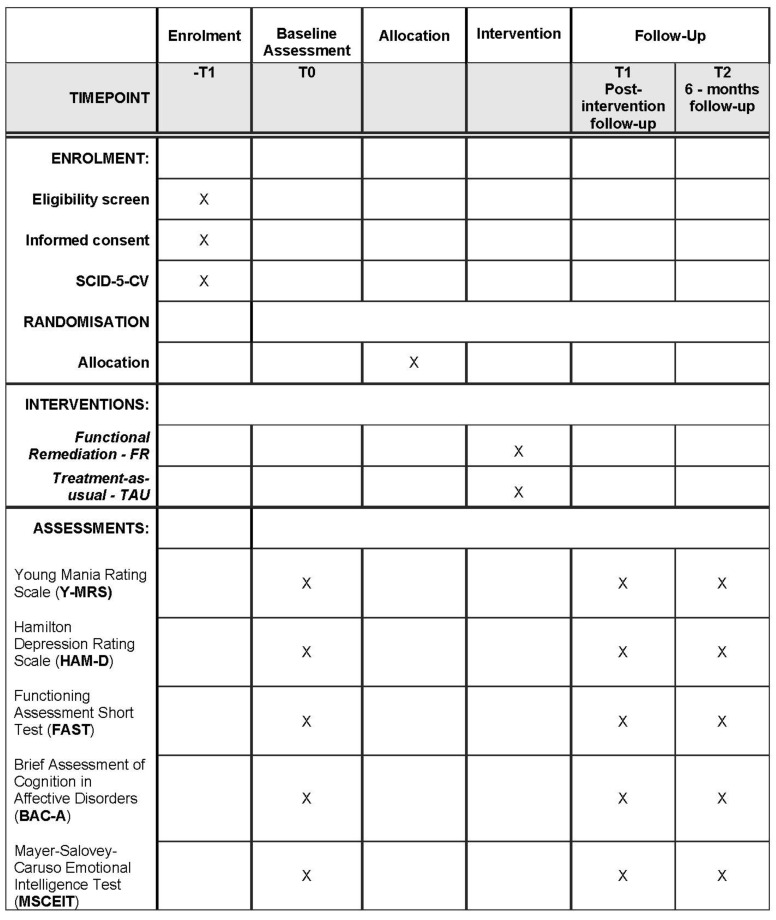
Schedule of enrolment, interventions and assessments. Brief Assessment of Cognition in Affective Disorders—BAC-A; functional remediation—FR; Functioning Assessment Short Test—FAST; Hamilton Depression Rating Scale—HAM-D; Mayer-Salovey-Caruso Emotional Intelligence Test—MSCEIT; Structured Clinical Interview for DSM-5 Disorders, Clinical Version—SCID-5-CV; treatment as usual—TAU; Young Mania Rating Scale—Y-MRS.

The enrolment phase will last six months. A systematic recruitment strategy will be applied, targeting all patients diagnosed with BD and receiving outpatient care. Eligible subjects will be identified through clinical documentation systems and informed of the study by their treating psychiatrist; they will be then approached by a study investigator to further discuss the study procedures. Only patients giving their consent will be administered the screening interview (shown in Figure 2).

#### 2.4.2. Allocation and Blinding

Patients fulfilling all inclusion criteria will be randomized to the two parallel groups by a researcher not involved in the subsequent trial procedures, using a computer-based system (1:1 ratio). The allocation sequence will not be based on any stratification procedure, and all the details related to randomization will be kept locked following treatment allocation (Figure 1).

#### 2.4.3. Intervention Phase

##### Functional Remediation

All study participants will continue receiving their usual treatment, and their service use will be documented. In addition, the intervention arm will receive FR, a manualized, group-based neurocognitive behavioral training program based on ecological tasks adapted to real-world situations [57]. The program is strongly focused on the development of cognitive strategies and their transfer to everyday situations, involving modeling techniques, role-playing tasks, self-instruction, positive reinforcement and metacognitive cues. These elements will be combined with psychoeducation sessions and with homework material assigned and discussed during each training session.

The program consists of 21 90-minute weekly sessions, in which a trained therapist and co-therapist work with 10–12 patients. The first 3 sessions cover psychoeducation on neurocognitive deficits, followed by 13 sessions of neurocognitive training sequentially targeting different cognitive domains (attention, memory and executive functions) and comprising paper-and-pencil cognitive exercises carried out either individually, in pairs or in small groups. Lastly, 5 sessions are dedicated to aspects of skills training, such as autonomy, communication, interpersonal relationships and stress management. Treatment will be administered by mental health professionals technically experienced in clinical neuropsychology, leading group therapies and BD care, identifying psychologists as main therapists and other professionals as co-therapists. One psychologist (V.A.) received direct specific training in the delivery of FR by the team of Barcelona Bipolar Disorders and Depressive Unit, Institute of Neurosciences, University of Barcelona, IDIBAPS, CIBERSAM, Hospital Clínic of Barcelona, who developed the program. Staff training will be conducted by V.A. before study start. Therapists and treatment teams will not take part in screening and outcome evaluations and will not disclose the treatment condition.

### 2.5. Treatment as Usual

Subjects enrolled in the TAU group will receive the prescribed pharmacological treatment according to the guidelines and good clinical practices envisaged for BD [50]. Participants recruited to the TAU group will not receive FR intervention.

Subjects enrolled and belonging to both groups will have access to other evidence-based treatments for bipolar disorder, according to clinical decisions.

### 2.6. Assessment Phase

Outcomes will be evaluated at baseline (T0), at the end of active treatment phase (T1) and 6 months thereafter (T2), using standardized and previously validated rating instruments based on Italian normative data. Assessments will be conducted by two trained residents in psychiatry, with at least 1 year of experience, not involved in treatment delivery for this trial and blinded to study treatment condition. Inter-rater reliability will be verified before study start. Patients will be instructed not to reveal information on their therapy during assessments. Eventual cases of blinding violations will be reported. Effectiveness of blinding will be verified at the end of the trial, asking assessors to guess which group the patients belong to and then verifying the answers using the McNemar test of independence [58]. Assessment at T1 is meant to obtain effectiveness data, together with essential information on trial and intervention feasibility. A follow-up observation will be performed to verify the long-term persistence of effects and their translation to everyday functioning, which is hypothesized to consolidate over time [59,60] (Figure 2).

### 2.7. Outcome Measures

The primary outcome for this study is the efficacy of FR in improving psychosocial functioning, measured using the FAST [56]. This is a simple rater-administered instrument specifically developed for patients with BD and comprising 24 items on various aspects of everyday functioning among those more frequently impaired. The FAST global score (0–72) reflects the patient’s level of disability. This scale has been consistently used in FR trials and already proven to be sensitive to effects of this treatment [39].

Additional outcomes will be *evaluated as secondary endpoints:* (a) global neurocognitive performance measured with the Brief Assessment of Cognition in Affective Disorders (BAC-A), Italian version [61]. This scale has been derived from a neuropsychological battery commonly used to assess cognitive performance in people living with schizophrenia, the Brief Assessment of Cognition in Schizophrenia (BACS) [62], but is designed to be sensitive and specific in patients with affective disorders such as BD. It contains six tests that assess different cognitive domains (List Learning for Verbal Memory, Digit Sequencing Task for Working Memory, Token Motor Task for Motor Speed, Verbal Fluency Category Instances for Semantic Fluency and Controlled Oral Word Association Test for Letter Fluency, the Tower of London Test for Executive Functions and Symbol Coding for Attention and Motor Speed) that are contained in the BACS and features also an affective processing test [63]. The six core BACS measures can be standardized into z-scores according to available normative data [61], and a global cognition composite score can be calculated by averaging the scores of single scales. (b) Socio-cognitive performance measured with the Mayer–Salovey–Caruso Emotional Intelligence Test (MSCEIT), Italian version [63]: this test assesses emotional processing ability and includes a total of 141 items divided in 8 tasks. The score on these tasks can be combined into 4 sub-scales that can be further combined in 2 areas and with a total score measuring global emotional intelligence. (c) Manic symptoms severity measured with the Y-MRS [54], a scale composed of 11 items. Four core items are graded from 0 to 8 without odd scores, and seven items are graded from 0 to 4; each item has specific anchor points, and higher scores reflect more severe manic symptoms. (d) Depressive symptoms severity measured with the HAM-D [55], a scale composed of 17 items. Each item has specific anchor points; 8 items can be graded from 0 to 2 and 9 items can be graded from 0 to 4, with higher scores reflecting greater symptoms severity; a score ≤ 7 corresponds to the absence of depressive symptoms.

The number and patterns of dropouts will also be reported, along with descriptions of the rate of enrollment and consent of eligible subjects, the percentage of session attendance and the reasons for non-attendance.

Patients will be considered dropouts in the following cases: (a) withdrawal of consent; (b) emergence of a full-blown mood episode and/or major change in drug therapy needed in order to prevent this eventuality within the study period; (c) discontinuation of drug therapy for ≥5 consecutive days; (d) missing ≥5 consecutive FR sessions.

Additional information will be collected regarding demographic and clinical characteristics of the included participants: age, sex, employment status, familiarity for psychiatric disorders, BD type I/II, cycling, seasonality, age at onset, duration of illness, number of previous episodes, history of psychosis, history of suicide attempts, presence of baseline residual symptoms and baseline cognitive performance (BAC-A), current therapy (baseline medications, changes in medication within the study period, lithium (yes/no), antipsychotics (yes/no)) and global daily drug burden, estimated using the WHO defined daily dose (DDD) method [64].

## 3. Measurement

### Statistical Analyses

All the analyses will be carried out using SPSS version 14.0 for Windows (SPSS Inc., Chicago, IL, USA, 2005).

Improvements in psychosocial functioning and cognitive performance will be assessed using effect size estimates obtained from within- and between-group differences at repeated measures analyses of variance (rm-ANOVAs). In case of attrition rate > 20%, a mixed model will be employed, under the missing at random (MAR) assumption. Regarding clinical symptoms, treatment effects will be compared among patients with and without residual symptoms (≥1 on Y-MRS, ≥3 on HAM-D) using t-tests.

Given the small sample size, the investigation of potential predictors of response through regression analyses should be considered largely explanatory. However, some regression analyses will be performed as deemed important in light of the feasibility nature of this trial; variables of interest will include participants’ age, duration of illness, baseline functioning, baseline cognitive performance and change in cognitive performance as well as potentially emerging predictors of dropout. In fact, participants leaving the study early will be compared with those completing all study procedures in terms of demographic and clinical variables using inferential statistics to assess potential moderators.

#### Power—Sample Size Calculation

The required sample size was estimated based on power calculations performed on the software G*Power [65] and assuming an effect size of 0.25 on the ANOVA group × time interaction for the primary outcome (psychosocial functioning measured with the FAST scale). This effect size is in line with that observed in previous studies adopting similar procedures and intervention paradigm [39]. With alpha at 0.05, the effect size could be detected with 95% power when enrolling a sample of 44 participants (22 per group). Assuming 20% attrition, it was decided to set the recruitment target at 54 participants (27 per group).

## 4. Discussion

The main aims of the present work are to assess the efficacy of FR for euthymic BD in improving psychosocial functioning and non-social and social cognitive performance.

The expected results are that FR could produce a positive effect on both the functioning and cognitive performance of participants, which could in turn have a consistent effect in improving the real-world, daily life functioning of participants, with positive implications also for their global quality of life [13].

In line with recent evidence, another expected result will be potential improvements of daily functioning related to changes in social cognition performance [66].

These results could replicate and further validate the available findings regarding the effectiveness of FR [39] and add to the growing amount of evidence indicating that CR-inspired interventions can produce substantial benefits in people living with BD [37], also considering the heterogeneity of the severity of cognitive impairments that characterize BD [32,33]. Obtaining a greater amount of evidence indicating that FR, as well as other CR-inspired interventions, represents an effective treatment for BD could lead to its inclusion into national and international treatment recommendations and foster a more consistent implementation of these interventions into clinical practice.

It is also possible that the findings of the present study could not support previous evidence indicating that FR is effective in improving functioning and cognition in BD. In this case, this negative finding would not detract from the efficacy of the intervention and its evidence-based nature but rather point out that some patient-, treatment- or setting-related characteristics could act as negative moderators of effectiveness, highlighting the need to further explore the role of these factors [57]. Several factors influenced the effectiveness and the acceptability of CR in trials including people living with schizophrenia [27,28,29,30,67], and in fact, studying the role of potential moderators of effectiveness also represents a secondary aim of the present study.

The present study shows a number of strengths. First and foremost, FR represents a psychosocial intervention that is characterized by relatively low resource requirements; it can be easily implemented in everyday rehabilitative practice in most clinical contexts. In fact, CR has been shown to produce significant improvements in people living with schizophrenia also in a real-world clinical context, using already available rehabilitation facilities [68,69]. Moreover, the assessment tools adopted in the present study represent a comprehensive and validated but simple and not excessively time-consuming array of instruments, including the BAC-A [63], which is currently recommended by the Cognition Task Force from the ISBD [8,38]. Both these factors could consistently contribute to support the evidence regarding the practical feasibility and implementation of the intervention but also positively contribute to the replicability of the observed findings.

However, some limitations have to be taken into account. Given the exploratory nature of the present study, the small sample size could represent the main limitation; in fact, while power analyses were conducted in order to define the recruitment of a sufficiently large sample to accurately evaluate the primary aim of the study, there is the possibility that secondary analyses—particularly those regarding potential moderators of response—could be underpowered. This fact will be taken into account both during data analysis and during the discussion of observed findings. Another limitation that has to be mentioned is that the FR-integrated intervention will be compared to TAU, which will not allow to infer potential comparisons between FR and other evidence-based psychosocial interventions validated in the treatment of BD. Cognitive screening, which is recommended in some of the available methodological recommendations [8,38], does not represent an inclusion criterion for the study; considering the cognitive heterogeneity of BD, this could lead to the inclusion of cognitively intact subjects in the trial, which could in turn reduce the chance of detecting treatment efficacy. However, it has also been hypothesized that people living with BD could benefit from the therapy regardless of their neurocognitive profile. Moreover, the use of FAST as a screening tool could help in detecting patients for whom neurocognitive impairment does actually interfere in everyday life [13]. Finally, diagnosis of neurological conditions represents an exclusion criterion for the present study, as they might affect the ability to perform neuropsychological tests beyond the impairments that can be commonly observed in people living with BD. However, given the possible high comorbidity between BD and some neurological conditions, such as multiple sclerosis [70,71,72], the issue of potential comorbidity should be explored more in detail, and future dedicated trials, including genetic testing, could be devised to further investigate these aspects.

Future research perspectives also include further exploring the cognitive heterogeneity of BD, leading to the development of target interventions that could be easily and practically adapted to the cognitive profile of each individual. This would represent an important improvement also considering the need to further develop personalized treatments and intervention strategies, a goal which is becoming more and more important in modern and personalized psychiatry and precision medicine [73]. From this perspective, better understanding the impact of moderators of effectiveness could play an important role. Moreover, a longitudinal observation of participants could allow to assess the durability of effects of the treatment and also provide further insight on the impact of treatment on the clinical and cognitive progression of the disorder.

Another interesting future development could be the investigation of the effectiveness of FR specifically in participants in the early or very early stages of the disease; interventions in this population could have a significant impact on the trajectory of the disorder, and establishing whether cognitive-oriented treatments could have a protective effect in longitudinal evaluations represents an important future step [74].

Better investigating neurobiological factors on the structural, functional and molecular levels represents another important area of future development [13,75,76,77,78]. In fact, furthering the knowledge and understanding of the neurobiological and neuropathological mechanisms involved in BD in the context of cognitive treatment could lead to the identification of both general and specific markers of response. This could allow a better standardization of treatment response monitoring as well as of illness trajectories, also providing valuable insight into the issue of potential cognitive decline in long-term BD patients [79,80,81].

Finally, if the results of the present study confirm the effectiveness of FR in BD, FR could be integrated into structured rehabilitation programs and combined with other non-pharmacological interventions targeting cognitive performance and aiming for functional improvement, such as physical exercise [82,83,84,85,86] or neurostimulation [85]. Establishing whether combined interventions provide greater or faster improvements, as has been observed in trials including participants diagnosed with schizophrenia [86,87,88], could provide valuable information for clinicians and mental health services.

In conclusion, this study could represent a valuable contribution to the research field, providing interesting information on the effectiveness of FR in BD. Considering both psychosocial functioning and cognitive performance as relevant outcomes, the results of this study could provide further groundwork for the development of evidence-based targeted and tailored interventions for people living with BD.

## Data Availability

Not applicable.

## Abbreviations

BAC-A: Brief Assessment of Cognition in Affective Disorders; BD: bipolar disorder; CR: cognitive remediation; FAST: Functioning Assessment Short Test; FR: functional remediation; HAM-D: Hamilton Depression Rating Scale; ISBD: International Society for Bipolar Disorders; MSCEIT: Mayer–Salovey–Caruso Emotional Intelligence Test; NICE: National Institute for Health and Care Excellence; SCID-5-CV: Structured Clinical Interview for Disorder for DSM-5, Clinical Version; TAU: treatment as usual; Y-MRS: Young Mania Rating Scale.
